# Notes on Preparations for the Sick

**Published:** 1895-06

**Authors:** 


### Notes on Preparations for the Sick.

**Vigoral. Extract of Beef. Nutrient Wine of Beef Peptone. Digestive Ferments.**—Armour & Co., London.—**Vigoral** makes a very pleasant and palatable beef-tea, and contains a good proportion of nutritious and stimulating constituents. The **Extract of Beef** we found to present the advantages claimed for it by the makers. The **Nutrient Wine of Beef Peptone** forms an excellent and easily assimilated food. The proteid constituents appear to be chiefly in the form of peptone, of which a large amount is present, with a small quantity of albumose. It is thus to be classed as a predigested food, suitable for all cases in which it is necessary to give food which can be assimilated without further effort on the part of the stomach. It is agreeable to take.

We tested the preparations of digestive ferments by artificial digestion mixtures, and found that speaking generally they had the properties claimed for them. **Lactated Pepsin,** with the addition of a little (·2 °/₀) HCl., readily converted fibrin into peptone, and starch into sugar. The **Glycerole Pepsin** acted energetically on fibrin, converting it into peptone, but not on starch. **Granular Pepsin** and **Powdered Pepsin** acted in the same way as the Glycerole Pepsin, but did not appear to be quite so potent. All these preparations might be relied upon in cases where stomach-digestion was at fault, and administration of the peptic ferment in some form was indicated. If the digestion of starch was also found to be weak, the first preparation should be the one selected.

**Pure Pancreatin** and **Glycerole Pancreatin** rapidly and completely digest all classes of food stuffs; the products formed being those normally present during pancreatic digestion. Both these preparations would be highly efficacious in the preparation of predigested foods; they are easily handled, give constant results, and the directions are simple and readily carried out.

We have said enough to show that in this series of digestive ferments Messrs. Armour & Co. provide in an elegant and convenient form very trustworthy aids to the treatment of the sick, and that the preparations are really what they are advertised to be.

---

**Hall's Coca Wine.**—Stephen Smith & Co., London.—The basis of this wine is an invalid's port, each two ounces of which contain the soluble constituents of one drachm of fresh Coca leaves. The tonic and invigorating effects of the Erythroxylon Coca are now well known, and this preparation gives us a standardised solution, which has been found to give excellent results in counteracting all kinds of mental and physical fatigue.

**Tabloids : Pepsin, Bismuth, and Charcoal ; Bismuth Salicylate ; Ferri, Quiniæ et Strychniæ Phosph. ; Salol ; Vin. Ipecac. ; Tinct. Camph. Co. ; Essence of Ginger ; Quinine Bisulphate.**—Burroughs, Wellcome and Co., London.—These Tabloids need no comment: their composition at once indicates their utility.

---

**Somnal** (Radlauer). **Antinervin.**—B. Kühn, London.—**Somnal,** the newest hypnotic, is said to be the most effective and most harmless of them all. It acts within half-an-hour, and the effect lasts from six to eight hours. The dose is from one-half to a teaspoonful, given in whisky, essence of ginger, syrup of liquorice, or syrup of raspberry. It is described as an "Ethylised Chloral Urethane," but it differs from chloral-urethane by the addition of $C_2 H_4$. It appears to combine the good qualities of the chloral hydrates and the urethanes, but without their unpleasant after-effects. It does not materially affect the blood-pressure, and does not give rise to digestive disturbances. Many physicians have reported excellent results from its use. In one case doses of thirty minims have given us excellent results, acting quickly and with no after-discomforts.

**Antinervin** is another substitute for antipyrine and phenacetin, a new and effective antipyretic and antineuralgic. It is a derivative of acetanilide, or rather a combination of two such derivatives, salicylanilide and bromanilide: it may be spoken of as salicyl bromanilide, or as it has been called by Dr. Frank Woodbury, sal-bromalide. In dealing with chronic neurotics we need a variety of such drugs as this : where one fails another may succeed.

---

The March number of this *Journal* contained (page 65) a report on some diabetic foods prepared by Messrs. G. Van Abbott & Sons, London. In a letter which we received from them shortly afterwards they stated their opinion "that there must be some mistake about the quantity of sugar found in the Caraway, Ginger, and Almond Cakes, as the flour used in the manufacture of them contains only a very small percentage of starch."

Accordingly, in response to their request, we have obtained a further analysis of these foods, and Mr. F. Wallis Stoddart, public analyst for Bristol, Salisbury and Bridgwater, reports to us that, after examination of the three varieties of food for diabetics made by Messrs. Van Abbott which were submitted to him, he does not find sugar in any. Starch is present only in insignificant quantity in the Caraway Biscuits and Almond Cakes, and is plainly attributable (as is shown by microscopic examination) to dusting the moulds with rice starch, a very common practice. The Ginger Biscuits contain an appreciable amount of starch, introduced, no doubt, with the powdered ginger.

On the other hand, all three foods yield very appreciable quantities of glucose when treated with dilute acids. The following were the proportions found:—

| | | |
|---|---|---|
| Caraway Biscuits ... ... ... | 18.0 per cent. |
| Ginger Biscuits ... ... ... | 19.2 | ,, |
| Almond Cake ... ... ... ... | 9.16 | ,, |

Mr. Stoddart regards this glucose as derived, not from starch or sugar, but from a mucilaginous carbohydrate such as commonly occurs in oily seeds of the kind from which the foods appear to be made. As it was necessary to exhaust the material of oil before attempting to invert, an approximate estimate of the amount gave the following results:—

| | | |
|---|---|---|
| Caraway Biscuits ... ... ... | 38.6 per cent |
| Ginger Biscuits ... ... ... | 36.5 | ,, |
| Almond Cake ... ... ... ... | 45.0 | ,, |

It is very probable that some of the glucose may be derived from cellulose also.

We regret that our previous report should have given a false impression as to the quantity of starch present in foods which are sold by the manufacturers as free from starch, but our further analysis still shows that these starch-free foods are capable of yielding considerable percentages of glucose. Whether the carbohydrate from which this glucose is derived is objectionable in the same degree as starch or sugar, is a question for the physician rather than the chemist. The general experience of diabetic patients tends to show that these foods are not capable of supplying an appreciable amount of sugar in the physiological laboratory of the human organism as they may be made to do in the test-tube of the chemist: we have often found no trace of sugar in the urine of patients so long as they adhere to their gluten bread and such diabetic foods, but the slightest transgression of the dietetic programme may be at once detected by an examination of the urine. Messrs. Van Abbott have done much to improve the prospects of the diabetic, and we would wish to avoid the appearance of casting any discredit on their manufactures.

